# The role of nurses, midwives, and doulas on breastfeeding: changes during the COVID-19 pandemic

**DOI:** 10.3389/fgwh.2025.1469428

**Published:** 2025-04-14

**Authors:** Shubhecchha Dhaurali, Shikhar Shrestha

**Affiliations:** ^1^ Department of Community Health, School of Arts and Sciences, Tufts University, Medford, MA, United States; ^2^ Epidemiology and Data Synthesis Unit, Center for Black Maternal Health and Reproductive Justice, Tufts University School of Medicine, Boston, MA, United States; ^3^ Department of Public Health and Community Medicine, Tufts University School of Medicine, Boston, MA, United States

**Keywords:** breastfeeding education, breastfeeding duration, COVID-19 pandemic, nurse, midwife, doula, PRAMS

## Abstract

**Introduction:**

The COVID-19 pandemic has significantly altered maternal healthcare delivery, including breastfeeding practices. Our study investigated the influence of nurses, midwives, and doulas on breastfeeding education and rates, with a specific focus on changes that transpired during the COVID-19 pandemic.

**Methods:**

Using a cross-sectional design, we performed a secondary data analysis on a stratified systematic sample of forty-six U.S. states and New York City respondents who completed the Pregnancy Risk Assessment Monitoring System (PRAMS) Phase 7 (2012–2015) and Phase 8 (2016–2020) surveys (*n* = 193,068). Descriptive analyses and adjusted multivariable logistic regression models reporting adjusted odds ratios (aORs) and 95% confidence intervals (95% CIs) were used to assess associations between the provision of breastfeeding guidance to mothers from nurses, doulas, or midwife healthcare professionals; breastfeeding/pumping rates; and the COVID-19 pandemic. Cox-proportional hazard models were used to examine the association between breastfeeding guidance and breastfeeding duration.

**Results:**

Our findings revealed that participants who received breastfeeding guidance from nurses, midwives, or doulas were twice as likely to have engaged in breastfeeding or milk pumping for their infants than participants who did not receive breastfeeding education (aOR = 1.99, 95% CI: 1.89–2.11, *p* < 0.0001). Additionally, participants who gave birth during the COVID-19 pandemic were notably less likely to receive breastfeeding education from a nurse, midwife, or doula than were those who gave birth before the pandemic (aOR = 0.92, 95% CI: 0.88–0.96, *p* < 0.0001). We also find that the hazard of stopping breastfeeding was lower among participants who received breastfeeding guidance (HR = 0.94, 95% CI: 0.91–0.97, *p* < 0.0001). Additionally, the hazard of stopping breastfeeding was lower during COVID-19 (HR = 0.94, 95% CI: 0.91–0.97, *p* = 0.001)

**Discussion:**

Our study underscores the vital role that healthcare professionals play in educating, advocating for, and promoting breastfeeding behaviors. This further highlights the pressing need for sustained efforts to support breastfeeding initiatives and address disparities in maternal and child health, particularly in the context of the challenges presented by the COVID-19 pandemic.

## 1 Introduction

The COVID-19 pandemic has had a profound influence on health and healthcare delivery, with the effects strongly felt by both pregnant and post-partum people (1, 2). Researchers have well-documented negative perinatal care experiences related to COVID-19 infection, visitor restrictions during labor/delivery and postpartum care, and lack of access to breastfeeding support (3). Labor and delivery nurses frequently report decreased time at the bedside during the COVID-19 pandemic, reducing the time they are able to spend with people during labor and immediately after delivery (4). Additionally, reduced/missed care has been reported within maternity settings and is associated with poorer practices, lower nursing support staff, and increased burnout, leading to lower rates of planned follow-up visits, adverse birth outcomes, and lower rates of exclusive breastfeeding rates (5). In fact, there has been a significant decline in the percentage of infants breastfed in the hospital during the pandemic (6), as well as breastfeeding support from hospitals and communities (7, 8).

Support from close family as well as healthcare providers is essential during the prenatal and postpartum periods as doulas and midwives positively influence breastfeeding rates and experiences (9–11). Doulas positively impact breastfeeding initiation and rates by serving as birthing individuals' peer counselors, offering emotional support, educating individuals on positive breastfeeding practices, and supporting their clients wherever and whenever they need (12). Mothers who are supported emotionally by doulas report lower stress and anxiety levels during labor and delivery, as well as improved and continued breastfeeding (9). Furthermore, when breastfeeding support is provided by a midwife, breastfeeding duration, and exclusivity increase, as midwives serve not only as breastfeeding experts (10), but also as emotional companions and friends (11).

The pandemic substantially altered breastfeeding rates and education, leading to adverse health outcomes and experiences for birthing people. Approximately 27% of mothers had difficulties obtaining help and faced various obstacles resulting from the implementation of lockdown measures, leading some to discontinue nursing prematurely (13). The pandemic resulted in restricted access to breastfeeding support; additionally, the interaction between a lactation expert and attending breastfeeding classes or support groups was reduced, resulting in decreased breastfeeding satisfaction overall (7). Such changes in breastfeeding care and education have especially affected Black birthing individuals and their newborns, who experience greater disparities in breastfeeding than all other groups. Specifically, Black birthing individuals have the lowest rates of breastfeeding initiation and duration (14–16), and racism is a contributing factor for reduced rates of breastfeeding initiation (17). Compared to non-Hispanic White women, non-Hispanic Black and Hispanic mothers were more likely to report racial discrimination in hospital settings as the cause of experiencing compounded stressors (18, 19), which play a significant role in their ability and desire to breastfeed (20).

A significant gap in the current research landscape is the limited exploration of how the COVID-19 pandemic has evolved the role of healthcare professionals, specifically nurses, midwives, and doulas, in educating birthing individuals on breastfeeding and the subsequent effects on breastfeeding rates/outcomes. Our study fills this gap through our prepandemic and pandemic analyses of the provision of breastfeeding education to mothers by maternity healthcare professionals, nurses, doulas, and midwives. Furthermore, we seek to understand how this result relates to breastfeeding and milk pumping rates. We hypothesize that doula and midwife-driven breastfeeding guidance is associated with increased rates of breastfeeding. In addition, we hypothesize that COVID-19 led to reduced doula-midwife interaction with birthing people leading to a reduction in breastfeeding initiation.

## 2 Methods

### 2.1 Data source

Founded in 1987 by the Centers for Disease Control and Prevention (CDC), the Pregnancy Risk Assessment Monitoring System (PRAMS) serves as a national population-based surveillance system with the primary objective of assessing risk factors related to maternal and infant morbidity and mortality to inform policies and interventions that can help reduce these adverse health outcomes. This system represents the singular nationwide surveillance mechanism that compiles extensive data concerning maternal well-being and experiences encompassing the prenatal, perinatal, and postpartum periods (21).

Operating across nearly all 50 U.S. states, including New York City, and territories of Puerto Rico and the District of Columbia, PRAMS covers approximately 83% of total U.S. births. Each participating state employs a stratified systematic sampling method, selecting 100–250 new mothers monthly from a pool of eligible birth certificates (22, 23). PRAMS respondents are birthing individuals and their infants who are sourced from the birth certificate file of the respective PRAMS participating state. Participants are then contacted through mail and telephone interviews for full survey completion (22, 23). For comprehensive insight into the study design and sampling methodologies of PRAMS, detailed information is available elsewhere (21).

### 2.2 Analytic samples

Our study sample consisted of PRAMS Phase 7 (2012–2015) and Phase 8 (2016–2020) participants across 46 states and New York City (only New York State, Arizona, and Arkansas are not represented in these phases); Sites were selected due to their inclusion of questions based on our selected exposure and outcome measures (24, 25). In addition to breastfeeding (*N* = 166,351) and income (*N* = 169,124), complete data were available for 89% of the sample (*N* = 193,068). To ensure the robustness of our analysis, we performed sensitivity analyses to impute data for participants who were missing ≥5% of demographic and outcome variable information. Both the national PRAMS and its respective site grantees provided the necessary authorization for the execution of this research. This study received approval from the Tufts University Institutional Review Board (STUDY #4141) and was classified as Non-Human Subjects Research.

### 2.3 Outcome measures

#### 2.3.1 Ever breastfed or pumped (breastfeeding rate)

We determined breastfeeding experience through the PRAMS core question, “Did you ever breastfeed or pump breast milk to feed your new baby, even for a short period of time?”. Participants who responded “yes” were coded affirmatively, and those who responded “no” were coded negatively.

#### 2.3.2 Breastfeeding education source nurse, midwife, or doula

The number of participants who received breastfeeding education from a nurse, midwife, or doula was determined via the core question, “Before or after your new baby was born, did you receive information about breastfeeding from… a nurse, midwife, or doula?” Participants who responded “yes” were coded “yes,” and those who responded “no” were coded “no.”

#### 2.3.3 Breastfeeding duration

The duration of breastfeeding reported by participants was determined through core question 37: “How many weeks or months did you breastfeed or feed pumped milk to your baby?” Participants could provide a written response, indicate if they never breastfed, or note if they were currently breastfeeding. In PRAMS, a calculated variable, “BF5WEEKS” estimated the duration of breastfeeding or continued breastfeeding status. We recoded the B5WEEKS variable to calculate the number of breastfeeding weeks for people still breastfeeding at the time of the interview using the date and month of birth and interview. We then created a censoring variable to indicate continued breastfeeding.

### 2.4 Exposure

#### 2.4.1 COVID-19 pandemic

We assessed the influence of the pandemic on the sources of breastfeeding education both before (2012–2019, *N* = 133,403) and during the pandemic (2020, *N* = 32,948). This measure was employed as a pivotal indicator to gauge potential shifts in the provision and reception of breastfeeding-related guidance and who provided such guidance to participants.

### 2.5 Data analysis

We created two-way frequency tables for our outcome variables and covariates by COVID-19 period (post vs. during). Furthermore, we constructed two-way frequency tables with chi-square tests to assess variations in the prevalence of maternal characteristics. To reduce the probability of observing false positive associations, we adjusted the significance level to *p* < 0.01. To investigate disparities in breastfeeding education received by a nurse, midwife, or doula before and during the pandemic, an adjusted multivariable logistic regression model was employed. We included covariates in the final model if the association was significant at *p* < 0.01 in the bivariate analysis. The final list of covariates encompassed a range of factors, including maternal age, education, marital status, race/ethnicity, household income, prenatal health insurance, parity, and pregnancy intention. We also examined the duration of breastfeeding among those who breastfeed based on breastfeeding education status and COVID-19 year, adjusting for other covariates identified in the models before. Since a large proportion of participants reported current breastfeeding at the time of the interview, we used the Cox proportional hazard model to account for the censoring of data. To verify our results, we imputed missing variables and conducted sensitivity analyses, and used survey weights to generate our estimates. The statistical analysis was performed using Stata Corp, LLC Version 18.0, and the final survey weights were appropriately integrated to ensure accurate representation of the results.

## 3 Results

### 3.1 Effects of the COVID-19 pandemic on breastfeeding rates

In our study, we included 183,014 study participants representing 153,706 births between 2012 and 2019 and 39,717 study participants representing 33,704 births in the year 2020 (Table 1). Breastfeeding rates increased with age, higher educational attainment, and higher household income both before and during the COVID-19 pandemic. Married participants reported higher breastfeeding rates than participants who were not married both before and during the pandemic (92% vs. 80%, *p* < 0.0001). Participants with private health insurance (92.9% and 93.6%) reported higher breastfeeding rates than participants with Medicaid (80.2% and 80.3%) or no insurance (86.3% and 84.7%) before and during the pandemic (*p* < 0.0001). Participants who intended their pregnancy reported higher breastfeeding rates than participants with unsure and unintended pregnancies before the pandemic (90.1% vs. 81.5% vs. 86.7%, *p* < 0.0001) and during (89.1% vs. 82.6% vs. 87.6%, *p* < 0.0001). Individuals with more than two live births had lower rates of breastfeeding than individuals with one or no live births both before (83.5% vs. 87.2% vs. 90.4%) and during (83.8% vs. 87.4% vs. 90.1%) the pandemic (*p* < 0.0001). The non-Hispanic Black participants reported the lowest breastfeeding rate before (78.2%) and during (76.6%) the pandemic (*p* < 0.0001). Non-Hispanic Asian participants reported higher breastfeeding rates both before and during the pandemic (93.4% vs. 93.3%, *p* < 0.0001).

**Table 1:** Prevalence of maternal demographics and breastfeeding rates in patients with COVID-19.

Variable	Before COVID-19 pandemic (2012–2019) (*N* = 153,706)		During COVID-19 pandemic (2020) (*N* = 33,704)	
	Total (%)	Ever breastfed or pumped (%)	Total (%)	Ever breastfed or pumped (%)
Total sample	82.2%	87.4%	17.8%	87.5%
Age (in years)	**	**	**	**
≤19	4.6%	78.5%	4.0%	74.5%
20–24	19.2%	83.3%	17.8%	82.4%
25–34	58.3%	88.7%	59.0%	88.9%
≥35	18.0%	89.7%	19.1%	90.7%
Education	**	**	**	**
<High school diploma	12.1%	77.6%	10.8%	77.8%
High school diploma	24.7%	80.2%	26.2%	79.6%
Some college	27.0%	87.5%	25.9%	88.3%
Completed college	36.2%	95.3%	37.1%	95.2%
Marital status	*	**	*	**
Other	39.3%	80.1%	40.5%	80.6%
Married	60.7%	92.1%	59.5%	92.1%
Race/ethnicity	**	**	**	**
Non-hispanic white	58.8%	88.2%	57.4%	89.3%
Non-hispanic black	15.8%	78.2%	16.1%	76.6%
Hispanic	18.7%	91.2%	20.1%	89.9%
Non-hispanic American Indian/Alaskan native	1.3%	85.4%	1.3%	84.1%
Non-hispanic Asian	3.9%	93.4%	3.9%	93.3%
Non-hispanic other	1.6%	91.1%	1.1%	90.9%
Household income^†^	**	**	**	**
$0–$24,000	33.2%	79.2%	29.9%	79.4%
$24,001–$48,000	19.6%	87.8%	19.1%	87.6%
$48,001–$73,000	13.1%	91.6%	14.2%	91.0%
>$73,000	34.2%	94.4%	36.8%	94.6%
Prenatal health insurance		**		**
Private	49.0%	92.9%	48.8%	93.6%
Medicaid	35.3%	80.2%	36.2%	80.3%
None	15.7%	86.3%	15.1%	84.7%
Pregnancy intention		**		**
Intended	43.9%	90.1%	44.6%	89.1%
Unintended	40.7%	86.7%	39.8%	87.6%
Unsure	15.4%	81.5%	15.6%	82.6%
Parity		**		**
None	39.0%	90.4%	39.6%	90.1%
One	33.0%	87.2%	32.8%	87.4%
Two or more	28.0%	83.5%	27.6%	83.8%
Breastfeeding education source nurse, midwife, or doula^††^	*	**	*	**
No	25.1%	81.1%	26.3%	81.9%
Yes	74.9%	89.7%	73.7%	89.5%

2012–2019 ^†^Income (*N* = 136,880); ^††^Breastfeeding Education (*N* = 132,981); 2020 ^†^Income (*N* = 29,023); ^††^Breastfeeding Education (*N* = 32,860).

* *p* < 0.01.

** *p* < 0.0001.

### 3.2 Effects of the COVID-19 pandemic on breastfeeding education rates

Participants who gave birth during the pandemic had significantly lower odds of receiving breastfeeding education from a nurse, midwife, or doula than did those who gave birth before the pandemic (aOR = 0.92, 95% CI = 0.88–0.96, *p* < 0.0001) (Table 2). Participants with a household income of more than $73,000 had significantly greater odds of receiving breastfeeding education than participants with a household income of $0–$24,000 both before (aOR = 1.27, 95% CI = 1.21–1.34, *p* < 0.0001) and during (aOR = 1.28, 95% CI = 1.07–1.32, *p* < 0.0001) the pandemic. Compared to Non-Hispanic White participants, Non-Hispanic Asian participants were more likely to receive breastfeeding education from a nurse, midwife, or doula (aOR = 1.15, 95% CI = 1.03–1.28, *p* < 0.01) in the pre-pandemic period. During the pandemic, non-Hispanic Asians had significantly greater odds than non-Hispanic whites (aOR = 1.53, 95% CI = 1.27–1.85, *p* < 0.0001). Non-Hispanic Black (aOR = 1.13, 95% CI: 1.02–1.25) and Hispanic participants (aOR = 1.12, 95% CI: 1.01–1.25) were also more likely to receive breastfeeding education from a nurse, midwife, or doula during the pandemic, but these associations were not significant enough to meet our threshold. Both before the pandemic and during the pandemic, participants with one previous live birth (aOR = 0.80, 95% CI = 0.76–0.84 and aOR = 0.87, 95% CI = 0.79–0.95, *p* < 0.0001) or two or more previous live births (aOR = 0.75, 95% CI = 0.71–0.79 and aOR = 0.71, 95% CI = 0.65–0.79, *p* < 0.0001) (a combination of one or two or more) were significantly less likely to receive breastfeeding education than participants with no previous live births (none). Participants with intended pregnancies were slightly more likely to receive education before the pandemic (aOR = 1.05, 95% CI = 1.00–1.09). This association became more significant during the pandemic. Participants with an intended intention had significantly greater odds (aOR = 1.20, 95% CI = 1.10–1.30, *p* < 0.0001) of receiving breastfeeding education from a nurse, midwife, or doula than participants with unintended pregnancy intention.

**Table 2:** Bivariate association between receiving breastfeeding education from a nurse, midwife or doula stratified by COVID-19 Status.

Variable	Breastfeeding education rates	
	OR (95% CI)	
During COVID-19 pandemic (2020)		
No	Reference	
Yes	0.92* (0.88–0.96)	
	Before COVID-19 Pandemic (2012–2019) (*N* = 181,875)	During COVID-19 Pandemic (2020) (*N* = 39,393)
Age (in years)		
≤19	Reference	
20–24	0.98 (0.88–1.1)	1 (0.81–1.24)
25–34	1.02 (0.92–1.14)	0.9 (0.74–1.1)
≥35	0.99 (0.88–1.1)	0.89 (0.72–1.1)
Education		
<High school diploma	Reference	
High school diploma	0.95 (0.88–1.02)	0.94 (0.81–1.09)
Some college	1.05 (0.98–1.13)	1.04 (0.9–1.19)
Completed college	1.28*** (1.2–1.37)	1.12 (0.98–1.29)
Marital status		
Other	Reference	
Married	1.07*** (1.03–1.11)	1 (0.92–1.08)
Race/ethnicity		
Non-hispanic white	Reference	
Non-hispanic black	1.05 (0.99–1.11)	1.13 (1.02–1.25)
Hispanic	1.03 (0.97–1.09)	1.12 (1.01–1.25)
Non-hispanic American Indian/Alaskan native	1.16 (1.03–1.31)	1.32 (1–1.74)
Non-hispanic Asian	1.15** (1.03–1.28)	1.53*** (1.27–1.85)
Non-hispanic other	1.2 (0.97–1.48)	1.1 (0.76–1.61)
Household income^†^		
$0–$24,000	Reference	
$24,001–$48,000	1 (0.94–1.06)	0.98 (0.87–1.11)
$48,001–$73,000	1.09 (1.02–1.17)	0.95 (0.83–1.08)
>$73,000	1.27*** (1.21–1.34)	1.19*** (1.07–1.32)
Prenatal health insurance		
Private	Reference	
Medicaid	0.89*** (0.85–0.93)	1.01 (0.93–1.09)
None	0.86*** (0.81–0.91)	0.91 (0.81–1.03)
Pregnancy intention		
Unintended	Reference	
Intended	1.05* (1.00–1.09)	1.20*** (1.10–1.30)
Unsure	0.86*** (0.81–0.91)	0.98 (0.87–1.09)
Parity		
None	Reference	
One	0.8*** (0.76–0.84)	0.87*** (0.79–0.95)
Two or more	0.75*** (0.71–0.79)	0.71*** (0.65–0.79)

^†^ Income (*N* = 161,528; *N* = 33,981).

* *p* < 0.05.

** *p* < 0.01.

*** *p* < 0.0001.

Prepandemic, participants who completed college were significantly more likely to receive breastfeeding education from a nurse, midwife, or doula than were those with less than a high school education (aOR = 1.28, 95% CI = 1.20–1.37, *p* < 0.0001). No such significant difference was found during the pandemic. Participants with Medicaid (aOR = 0.89, 95% CI: 0.85–0.93, *p* < 0.0001) or no insurance (aOR = 0.86, 95% CI: 0.81–0.91, *p* < 0.0001) were significantly less likely to receive breastfeeding education from a nurse, midwife, or doula than participants with private insurance before the pandemic. Married participants were significantly more likely to receive breastfeeding education from a nurse, midwife, or doula than participants who were unmarried (aOR = 1.07, 95% CI = 1.03–1.11, *p* < 0.0001). This association was not observed during the pandemic. Prepandemic, participants with unsure pregnancy intent had significantly lower odds (aOR = 0.86, 95% CI = 0.81–0.91, *p* < 0.0001) of receiving breastfeeding education from a nurse, midwife, or doula than participants with unintended pregnancy intention. This association was not observed during the pandemic. Our study conducted a comparison between the previously described findings and the outcomes derived from our sensitivity analyses employing multivariate logistic regression with imputed values, yielding consistent results. For further details, please consult Appendix Table A1.

### 3.3 Breastfeeding rates, COVID-19, breastfeeding education, and demographics

Participants who received breastfeeding education from a nurse, midwife, or doula had twice the odds of ever breastfeeding than participants who did not receive breastfeeding education from the mentioned maternity care sources (aOR = 1.99, 95% CI = 1.89–2.11, *p* < 0.0001) (Table 3). Participants were more likely to report having ever breastfed during the pandemic than before the pandemic (aOR = 1.40, 95% CI = 1.09–1.80, *p* < 0.01). We observed a significant interaction effect between no insurance and pregnancy on the odds of breastfeeding after controlling for other confounders (aOR = 0.74, 95% CI = 0.58–0.94, *p* < 0.01). Compared to participants without a high school education, those who completed college had almost four times the odds of ever breastfeeding (aOR = 3.78, 95% CI = 3.34–4.28, *p* < 0.0001). Non-Hispanic Black participants were significantly less likely to report ever breastfeeding than non-Hispanic White participants were (aOR = 0.78, 95% CI = 0.73–0.84, *p* < 0.0001). Hispanic participants were twice as more likely to ever breastfeed than non-Hispanic White participants were (aOR = 2.48, 95% CI = 2.27–2.71, *p* < 0.0001). For comparisons between these findings and the sensitivity analysis outcomes, please consult Appendix Table B1.

**Table 3:** Associations between breastfeeding rates; receiving breastfeeding education from a nurse, midwife, or doctor; and COVID-19 incidence (*n* = 146,383).

Variable	Ever breastfed or pumped OR (95% CI)
Breastfeeding education source nurse, midwife, or doula	
No	Reference
Yes	1.99** (1.89–2.11)
During COVID-19	
No	Reference
Yes	1.4* (1.09–1.8)
Insurance & COVID-19 interaction	
Medicaid#Yes	0.82 (0.69–0.99)
None#Yes	0.74* (0.58–0.94)
Marital status & COVID-19 interaction	
Married#Yes	0.9 (0.76–1.05)
Education & COVID-19 interaction	
High school#Yes	0.87 (0.7–1.07)
Some college#Yes	0.91 (0.73–1.13)
College completed#Yes	0.79 (0.6–1.03)
Age (in years)	
≤19	Reference
20–24	1.02 (0.9–1.16)
25–34	1.01 (0.89–1.16)
≥35	0.91 (0.78–1.05)
Education	
<High school diploma	Reference
High school diploma	1.27** (1.16–1.39)
Some college	1.94** (1.77–2.14)
Completed college	3.78** (3.34–4.28)
Marital status	
Other	Reference
Married	1.54** (1.44–1.66)
Race/ethnicity	
Non-hispanic white	Reference
Non-hispanic black	0.78** (0.73–0.84)
Hispanic	2.48** (2.27–2.71)
Non-hispanic American Indian/Alaskan Native	1.38** (1.19–1.59)
Non-hispanic Asian	1.47** (1.25–1.74)
Non-hispanic other	1.79** (1.31–2.46)
Household income	
$0–$24,000	Reference
$24,001–$48,000	1.48** (1.38–1.59)
$48,001–$73,000	1.61** (1.45–1.78)
>$73,000	1.77** (1.59–1.96)
Prenatal health insurance	
Private	Reference
Medicaid	0.9 (0.82–0.98)
None	1.16* (1.04–1.30)
Pregnancy intention	
Unintended	Reference
Intended	0.98 (0.92–1.04)
Unsure	0.9** (0.84–0.97)
Parity	
None	Reference
One	0.67** (0.62–0.71)
Two or more	0.62** (0.57–0.67)

* *p* < 0.01.

** *p* < 0.0001.

### 3.4 Breastfeeding duration, COVID-19, and breastfeeding education

Approximately 22% of study participants did not breastfeed, or their breastfeeding information was reported as don't know or missing. Of the people who reported breastfeeding, 28.7% had stopped breastfeeding at the time of the interview, and 71.3% reported breastfeeding at the time of the interview. Among the participants who had stopped breastfeeding, the average duration of breastfeeding was 6.6 weeks (95% CI: 6.5–6.7), and among participants who were still breastfeeding, the average duration of breastfeeding at the time of the interview was 16.8 weeks (95% CI: 16.7–16.8). The Cox proportional hazard model showed that participants who had received breastfeeding education from a nurse, midwife, or doula had a 6% lower hazard of stopping breastfeeding (HR: 0.94, 95% CI: 0.91–0.97) at any time compared to those who did not receive the breastfeeding education (Table 4). In addition, the hazard of stopping breastfeeding during the pandemic was 6% lower than in the pre-pandemic period (HR: 0.94, 95% CI: 0.91–0.97) (Table 4). Increasing age was associated with a lower hazard of stopping breastfeeding at any time (Table 4). Similarly, higher education was associated with a lower hazard of stopping breastfeeding at any time, with college-educated participants having a 50% lower hazard (HR: 0.5, 95% CI: 0.47–0.53) (Table 4). Non-Hispanic black participants had a higher hazard of stopping breastfeeding (HR: 1.1 95% CI: 1.09–1.17) compared to non-Hispanic whites, whereas all other race groups had a lower hazard of breastfeeding at any time (Table 4). Higher income was also associated with a lower hazard of stopping breastfeeding at any time (Table 4). Having Medicaid insurance was associated with a higher hazard of stopping breastfeeding at any time compared to private insurance (HR: 1.09, 95% CI: 1.05–1.14) (Table 4).

**Table 4:** Associations between breastfeeding duration; receiving breastfeeding education from a nurse, midwife, or doctor; and COVID-19 incidence.

Variable	Breastfeeding duration hazard ratio (95% CI)
Breastfeeding education source nurse, midwife, or doula	
No	Reference
Yes	0.94*** (0.91–0.97)
During COVID-19 pandemic (2020)	
No	Reference
Yes	0.94** (0.91–0.97)
Age (in years)	
≤19	Reference
20–24	0.91** (0.85–0.97)
25–34	0.86*** (0.81–0.92)
≥35	0.89** (0.83–0.96)
Education	
<High school diploma	Reference
High school diploma	1.02 (0.97–1.07)
Some college	0.84*** (0.8–0.88)
Completed college	0.51*** (0.48–0.54)
Marital status	
Other	Reference
Married	0.68*** (0.66–0.71)
Race/ethnicity	
Non-hispanic white	Reference
Non-hispanic black	1.13*** (1.09–1.17)
Hispanic	0.92*** (0.89–0.96)
Non-hispanic American Indian/Alaskan native	0.9** (0.83–0.96)
Non-hispanic Asian	0.83*** (0.77–0.9)
Non-hispanic other	0.78** (0.68–0.91)
Household income^†^	
$0–$24,000	Reference
$24,001–$48,000	0.86*** (0.83–0.89)
$48,001–$73,000	0.82*** (0.78–0.86)
>$73,000	0.74*** (0.7–0.78)
Prenatal health insurance	
Private	Reference
Medicaid	1.1*** (1.05–1.14)
None	0.92** (0.87–0.96)
Pregnancy intention	
Unintended	Reference
Intended	0.94*** (0.91–0.97)
Unsure	1.04 (1–1.08)
Parity	
None	Reference
One	0.89*** (0.86–0.92)
Two or more	0.81*** (0.78–0.84)

* *p* < 0.05.

** *p* < 0.01.

*** *p* < 0.0001.

## 4 Discussion

Our study emphasizes the critical role of healthcare professionals, specifically nurses, midwives, and doulas, in delivering essential breastfeeding care and education and in impacting breastfeeding rates before and during the pandemic. Participants who received breastfeeding education from nurses, midwives, or doulas were twice as likely to have ever breastfed or pumped milk than participants who did not receive such education. Our findings emphasize the critical role that nurses, midwives, and doulas play in encouraging mothers to breastfeed/pump milk as well as providing education on important breastfeeding practices. Furthermore, this study provides evidence for enacting policies and funding training programs that ensure that nurses, midwives, and doulas are equipped with the necessary knowledge and skills to support breastfeeding, inclusive of staying updated on the latest breastfeeding guidelines and techniques. Additionally, we found that participants who gave birth during the COVID-19 pandemic were significantly less likely to receive breastfeeding education from a nurse, midwife, or doula than participants who gave birth before the pandemic. Our findings highlight the pandemic's significant consequence on access to breastfeeding education, underscore the need for public health preparedness, and suggest the potential value of telehealth/virtual health support platforms to connect patients and providers during crises such as the pandemic to offer breastfeeding care.

Approximately three-quarters of the participants were educated about breastfeeding by a nurse, midwife, or doula both before and during the pandemic. Breastfeeding rates were approximately 90% across both periods. Participants who received breastfeeding guidance from nurses, midwives, or doulas were twice as likely to have ever breastfed or pumped milk to their infant than participants who did not receive education. The literature consistently recognizes nurses, midwives, and doulas as vital sources of breastfeeding information and support (9–11). One study reported nurses, midwives, and doulas to be the second most prevalent source of breastfeeding information after a doctor (16). Our findings add to the existing evidence supporting the importance of nurses, midwives, and doulas in providing breastfeeding education and rates and provide evidence to lawmakers and hospital systems to recognize the significance of such healthcare professionals in promoting breastfeeding and considering strategies to enhance their involvement and training.

Moreover, our study revealed that participants who delivered their babies amidst the COVID-19 pandemic exhibited a notable decrease in the likelihood of receiving breastfeeding guidance from healthcare professionals, such as nurses, midwives, or doulas, in contrast to those who gave birth prior to the pandemic. Possible explanations for decreased breastfeeding education during the pandemic explored in previous literature included large-scale shifts in maternity care, fear of COVID-19 transmission/illness, lack of care coordination, and consistency in support (3, 26, 27). Specifically, the healthcare practices during childbirth and during the postpartum period that significantly changed during the pandemic included the employment of visitor restrictions, not permitting birthing support (doulas, birth companions) during delivery/postpartum, and separating the newborn from the parent without breastfeeding support (3). Another change in healthcare during the pandemic was the shift to telehealth and virtual care for provider-patient perinatal meetings/appointments (28, 29). In one study, doulas reported a loss of connection in their role as advocates when moving care virtually, difficulties in helping patients switch to virtual care (30), and worsening breastfeeding disparities for patients without access to lactation support and care (31). Although there were several barriers to providing care for birthing and nursing mothers during the COVID-19 pandemic, published reports doulas and other maternity care workers “were willing to go the extra mile” to aid their patients (31).

We found that participants were more likely to report having breastfed or pumped during the pandemic than before the pandemic contrary to our hypothesis. Our finding does not align with studies reporting a decrease in breastfeeding rates and support during the pandemic (6, 8). However, these findings align with the literature reporting that COVID-19 stay-at-home orders delay breastfeeding cessation plans (32). Recent literature separating the pandemic into early and late periods concluded that mothers during the early pandemic period received less support and education due to the novelty and confusion caused by the coronavirus and its significant effect on hospital systems compared to mothers during the pre- and late pandemic periods (7). Once telehealth was initiated, breastfeeding resources became more accessible for participants. Overall, the study concluded that, compared to prepandemic parents, both early- and late-pandemic parents had lower odds of meeting their breastfeeding goals (7). Furthermore, many mothers reported that a lack of or decreased social and professional breastfeeding support significantly impacted their breastfeeding experience(s) during the pandemic (33).

Although non-Hispanic Asians made up less than four percent of our total prepandemic and pandemic samples, the group reported the highest breastfeeding rates compared to all other racial/ethnic group rates. Our findings are consistent with the literature reporting that Asian mothers have longer overall breastfeeding durations and initiation rates than mothers of other racial/ethnic groups (14, 34). In a recent study, Asian (93%) and Native Hawaiian (99%) women were more likely to report breastfeeding or breastfeeding longer than 10 weeks (16). However, in the previously mentioned study and on our own, disaggregation of the heterogeneous Asian population was not possible. A study before the pandemic that looked at distinct Asian populations revealed breastfeeding disparities within the groups (35). Due to the various, distinct cultures around breastfeeding in this group (and others), it is likely that the results do not reflect all populations classified as “Asian” in this study. Furthermore, non-Hispanic Asian participants were also more likely to receive breastfeeding education from a nurse, midwife, or doula (association increased in significance before the pandemic) and to report ever breastfeeding/pumping than their non-Hispanic White counterparts were. Our study also supports the findings of previous reports in the literature showing that providing breastfeeding information via nurses, midwives, and doulas to populations, including Asian women, is extremely beneficial (16).

Non-Hispanic Black participants made up approximately sixteen percent of both the prepandemic and pandemic samples, with slightly more than three-quarters of the population reporting breastfeeding in both samples. Compared to non-Hispanic White participants, non-Hispanic Black participants were significantly less likely to report breastfeeding. In fact, all the other racial groups except Non-Hispanic Black participants reported higher odds of ever having breastfed/pumped than did the non-Hispanic White participants. Our results are consistent with other published studies on the racial/ethnic disparities in breastfeeding among Black birthing people (14, 16). Additionally, approximately twenty percent of the Hispanic participants were from the two cohorts, and approximately ninety percent of the population reported breastfeeding. Hispanic participants were more than two times more likely to report breastfeeding than their White counterparts were. Our study results are consistent with the literature. Previous studies have reported that Hispanic mothers have higher levels of breastfeeding initiation and continuation than do all demographic populations; additionally, Hispanic mothers are more likely to intend to breastfeed than black mothers are, and Mexican American mothers are more likely to breastfeed than non-Hispanic white mothers (34, 36, 37). Interestingly, we found that both non-Hispanic Black and Hispanic participants were more likely to receive breastfeeding information from a nurse, midwife, or doula during the pandemic, although these associations did not meet our significance threshold. Our findings support the findings of previous studies showing the importance of racial/ethnic minorities, black women in particular, receiving breastfeeding education from nurses, midwives, and doulas in increasing breastfeeding rates (16, 38–40).

For both the pre- and pandemic periods, approximately half of our individuals were privately insured, with approximately thirty-five percent insured with Medicaid (government health insurance program for low-income adults and children (41) and the rest uninsured. Among these populations, the highest breastfeeding rate was among privately insured participants, and the lowest rate was among Medicaid-insured participants during both the prepandemic and pandemic periods. Our findings are consistent with the literature that showcase socioeconomic disparities in breastfeeding practices (42, 43). Compared to participants with private insurance during the prepandemic period, those with Medicaid or no insurance were significantly less likely to receive breastfeeding education from a nurse, midwife, or doula. We did not observe this association during the pandemic, although we did observe a significant interaction between having no insurance and the pandemic period, which lowered the odds of breastfeeding.

Approximately forty percent of our prepandemic and pandemic samples completed college, and slightly more than a tenth did not complete high school. Among the education levels, the highest percentage of breastfeeding (more than ninety-five percent) was reported by participants with college degrees, and the lowest was reported by participants who did not graduate high school both before and during the pandemic. Participants who completed college were significantly more likely to receive breastfeeding education from a nurse, midwife, or doula than participants with less than a high school education before the pandemic. Interestingly, we did not observe this association during the pandemic. Individuals with a high education level were significantly more likely to report ever breastfeeding than participants without a high school diploma were college-educated participants were almost four times more likely to breastfeed than participants without a high school diploma were.

Slightly less than half of our sample both during the pandemic and before the pandemic reported their pregnancy as intended, approximately forty percent unintended, and fifteen percent unsure. Among these populations and for both pandemic periods, the highest breastfeeding rate was among participants with intended pregnancies, and the lowest rate was among participants with unsure intent. Both before and during the pandemic, participants with intended pregnancies were more likely to receive breastfeeding education from a nurse, midwife, or doula, with the more significant association occurring during the pandemic.

Breastfeeding education reduced the risk of early cessation or reduced breastfeeding duration, emphasizing the importance of support and education for mothers during this critical period. Additionally, our analysis found that breastfeeding duration increased during the pandemic, most likely due to stay-at-home orders, which gave mothers more time to breastfeed. This is consistent with findings from a meta-analysis on breastfeeding initiation and continuation, which highlighted various factors influencing breastfeeding duration (32). Hamad et al. and Huang and Yang have long reported that breastfeeding duration tends to increase when mothers are given adequate time off, such as paid family leave or maternity leave (44, 45). These findings align with Hamad et al.'s 2023 findings that breastfeeding duration increased post-COVID-19 lockdowns (46). However, our study accounts for censoring of breastfeeding duration thereby providing an estimate of hazard of breastfeeding stoppage rather than average duration of breastfeeding, which is appropriate given the large censoring of the duration of breastfeeding variable in PRAMS.

### 4.1 Limitations

PRAMS data are self-reported and may be prone to recall biases as participants were surveyed about their prenatal, perinatal, and postpartum experiences 2–6 months after childbirth. Furthermore, PRAMS exclusively includes only participants with live births in their dataset, limiting the generalizability of findings from those who experienced fetal loss, terminated pregnancy, or did not have a live birth. The grouping of nurses, midwives, and doulas does not delineate their inter-complexities adequately. Pandemic-related restrictions have significantly affected doula practices, potentially biasing outcomes, especially considering their private hire status and lack of insurance coverage, limiting generalizability. Furthermore, PRAMS data are exclusively reported annually; thus, “2020” was classified in our study as occurring during the pandemic, although the pandemic was not declared a national emergency in the U.S. until March 13, 2020.

### 4.2 Strengths

Despite our limitations, our study also includes several strengths. One is our utilization of the extensive and representative national PRAMS dataset encompassing a diverse sample of postpartum individuals across the U.S. ensuring the reliability and comparability of the data across all participants. Furthermore, our large sample size enhances the generalizability and statistical power of our findings, allowing us to draw meaningful conclusions about the influence of the COVID-19 pandemic on breastfeeding information. In addition, in addition to sensitivity analyses imputing for missing data, our statistical analysis employed methods to control potential confounders, enhancing the reliability and overall robustness of our results. This study underscores the vital role of nurses, midwives, and doula healthcare professionals in promoting breastfeeding and highlights the disparities in breastfeeding rates among different racial and ethnic groups.

## 5 Conclusion

Our study provides critical insights into the impact of the COVID-19 pandemic on breastfeeding practices and healthcare support in the U.S. We observed a significant decrease in the provision of breastfeeding information provided by nurses, midwives, and doulas during the pandemic compared to before the pandemic. Furthermore, our study underscores the pivotal role of nurses, midwives, and doulas in promoting breastfeeding. Participants who received guidance from these professionals were more likely to initiate and continue breastfeeding. These findings also bring attention to the disparities in breastfeeding rates and support among different racial and ethnic groups. This study emphasizes the critical role of healthcare professionals in educating, advocating, and promoting breastfeeding behaviors and the need for continued efforts to support breastfeeding initiatives and address disparities in maternal and child health.

## Data Availability

Publicly available datasets were analyzed in this study. This data can be found here: https://www.cdc.gov/prams/php/data-research/arf-data-access-form.html

## Appendix

**Table A1:** Analysis of the association between receiving breastfeeding education from a nurse, midwife or doula stratified by COVID-19 and outcomes.

Variable	Breastfeeding education rates	
	OR (95% CI)	
During COVID-19 pandemic (2020)		
No	Reference	
Yes	0.92* (0.88–0.96)	
	Before COVID-19 Pandemic (2012–2019)	During COVID-19 Pandemic (2020)
Age (in years)		
≤19	Reference	
20–24	0.99 (0.88–1.1)	1 (0.81–1.24)
25–34	1.03 (0.92–1.14)	0.9 (0.74–1.1)
≥35	0.99 (0.88–1.10)	0.89 (0.72–1.1)
Education		
<High school diploma	Reference	
High school diploma	0.95 (0.88–1.02)	0.94 (0.81–1.09)
Some college	1.05 (0.98–1.13)	1.04 (0.90–1.19)
Completed college	1.28* (1.20–1.37)	1.12 (0.98–1.29)
Marital status		
Other	Reference	
Married	1.07* (1.03–1.11)	1 (0.92–1.08)
Race/ethnicity		
Non-hispanic white	Reference	
Non-hispanic black	1.05 (0.99–1.11)	1.13 (1.02–1.25)
Hispanic	1.03 (0.97–1.09)	1.12 (1.01–1.25)
Non-hispanic American Indian/Alaskan native	1.16 (1.03–1.31)	1.32 (1–1.74)
Non-hispanic Asian	1.15** (1.03–1.28)	1.53* (1.27–1.85)
Non-hispanic other	1.20 (0.97–1.48)	1.11 (0.76–1.61)
Household income		
$0–$24,000	Reference	
$24,001–$48,000	0.98 (0.93–1.04)	0.96 (0.86–1.08)
$48,001–$73,000	1.07 (1.00–1.14)	0.94 (0.83–1.06)
>$73,000	1.24* (1.17–1.30)	1.19** (1.04–1.26)
Prenatal health insurance		
Private	Reference	
Medicaid	0.89* (0.85–0.93)	1.01 (0.93–1.10)
None	0.86* (0.81–0.91)	0.91 (0.81–1.03)
Pregnancy intention		
Unintended	Reference	
Intended	1.05*** (1.00–1.09)	1.20* (1.10–1.30)
Unsure	0.86* (0.81–0.91)	0.98 (0.87–1.09)
Parity		
None	Reference	
One	0.80* (0.76–0.84)	0.87* (0.79–0.95)
Two or more	0.75* (0.71–0.79)	0.71* (0.65–0.79)

* *p* < 0.0001.

** *p* < 0.01.

*** *p* < 0.05.

**Table B1:** Analysis of the association between breastfeeding rates, receiving breastfeeding education from a nurse, midwife, or doula, and COVID-19 outcomes.

Variable	Ever breastfed or pumped OR (95% CI)
Breastfeeding education source nurse, midwife, or doula	
No	Reference
Yes	1.93* (1.83–2.03)
During COVID-19	
No	Reference
Yes	1.21 (0.95–1.53)
Insurance & COVID-19 interaction	
Medicaid#Yes	0.85 (0.64–0.71)
None#Yes	0.72** (0.58–0.90)
Marital status & COVID-19 interaction	
Married#Yes	0.93 (0.81–1.08)
Education & COVID-19 interaction	
High school#Yes	0.94 (0.78–1.14)
Some college#Yes	1.01 (0.83–1.24)
College completed#Yes	0.87 (0.68–1.12)
Age (in years)	
≤19	Reference
20–24	1.12 (1.00–1.26)
25–34	1.12 (1.00–1.26)
≥35	1.01 (0.89–1.17)
Education	
<High school diploma	Reference
High school diploma	1.31* (1.21–1.43)
Some college	1.97* (1.81–2.15)
Completed college	3.85* (3.42–4.33)
Marital status	
Other	Reference
Married	1.54* (1.44–1.66)
Race/ethnicity	
Non-hispanic white	Reference
Non-hispanic black	0.78* (0.73–0.84)
Hispanic	2.40* (2.21–2.61)
Non-hispanic American Indian/Alaskan native	1.47* (1.29–1.59)
Non-hispanic Asian	1.43* (1.25–1.67)
Non-hispanic other	1.83* (1.37–2.44)
Household income	
$0–$24,000	Reference
$24,001–$48,000	1.42* (1.32–1.51)
$48,001–$73,000	1.53* (1.39–1.68)
>$73,000	1.70* (1.54–1.88)
Prenatal health insurance	
Private	Reference
Medicaid	0.89** (0.82–0.98)
None	1.14** (1.03–1.27)
Pregnancy intention	
Unintended	Reference
Intended	0.98 (0.92–1.04)
Unsure	0.88* (0.83–0.95)
Parity	
None	Reference
One	0.68* (0.62–0.72)
Two or more	0.62* (0.58–0.67)

* *p* < 0.0001.

** *p* < 0.01.

*** *p* < 0.05.
